# Differential Diagnosis of Gastric Ulcer and Cancer

**Published:** 1881-01

**Authors:** 


Kaposi in *L'Union Medicale* gives the following ointment of lead and vaseline for squamous skin diseases:

R.—Emplastrum Plumbum........℥ ii
Vaseline........................℥ ii

M.—These are mixed at gentle heat and removed before cooling, and may be perfumed by bergamot, lavender, or Bals. Peru. The author recommends this ointment to detach the crusts and scales in certain skin diseases, and more particularly in eczema squamosum, when the skin is dry and covered with epidermic scales. Even on excoriated surfaces it does not occasion any sensation of burning, and is preferable to Hebra's plaster, because it is inodorous, and does not change when kept long in contact with the skin.—*American Medical Bi-Weekly.*

